# A novel deubiquitinase inhibitor b-AP15 triggers apoptosis in both androgen receptor-dependent and -independent prostate cancers

**DOI:** 10.18632/oncotarget.18774

**Published:** 2017-06-28

**Authors:** Jianyu Cai, Xiaohong Xia, Yuning Liao, Ningning Liu, Zhiqiang Guo, Jinghong Chen, Li Yang, Huidan Long, Qianqian Yang, Xiaolan Zhang, Lu Xiao, Xuejun Wang, Hongbiao Huang, Jinbao Liu

**Affiliations:** ^1^ Protein Modification and Degradation Lab, SKLRD, School of Basic Medical Sciences, The Affiliated Cancer Hospital of Guangzhou Medical University, Guangdong 511436, China; ^2^ Guangzhou Institute of Cardiovascular Disease, The Second Affiliated Hospital, Guangzhou Medical University, Guangzhou, Guangdong 510260, China; ^3^ Division of Basic Biomedical Sciences, Sanford School of Medicine of The University of South Dakota, Vermillion, South Dakota 57069, USA

**Keywords:** b-AP15, deubiquitinase inhibitor, prostate cancer, apoptosis

## Abstract

Prostate cancer (PCa) remains a leading cause of cancer-related death in men. Especially, a subset of patients will eventually progress to the metastatic castrate-resistant prostate cancer (CRPC) which is currently incurable. Deubiquitinases (DUBs) associated with the 19S proteasome regulatory particle are increasingly emerging as significant therapeutic targets in numerous cancers. Recently, a novel small molecule b-AP15 is identified as an inhibitor of the USP14/UCHL5 (DUBs) of the 19S proteasome, resulting in cell growth inhibition and apoptosis in several human cancer cell lines. Here, we studied the therapeutic effect of b-AP15 in PCa, and our results indicate that (i) b-AP15 decreases viability, proliferation and triggers cytotoxicity to both androgen receptor-dependent and -independent PCa cells *in vitro* and *in vivo*, associated with caspase activation, inhibition of mitochondria function, increased reactive oxygen species (ROS) generation and endoplasmic reticulum (ER) stress; (ii) pan-caspase inhibitor z-VAD-FMK and ROS scavenger N-acetyl-L-cysteine (NAC) efficiently block apoptosis but not proteasome inhibition induced by exposure of b-AP15; (iii) treatment with b-AP15 in androgen-dependent prostate cancer (ADPC) cells down-regulates the expression of androgen receptor (AR), which is degraded *via* the ubiquitin proteasome system. Hence, the potent anti-tumor effect of b-AP15 on both androgen receptor-dependent and -independent PCa cells identifies a new promising therapeutic strategy for prostate cancer.

## INTRODUCTION

Recent epidemiological data identify prostate cancer (PCa) as the second most frequently diagnosed cancer of men and the fifth most common cancer overall in the world [1]. The incidence of prostate cancer is increasing gradually in almost all countries [2]. While the five-year survival rate for local PCa is close to 100% due to the availability of curative treatments such as castration, the patients with invasive and metastatic disease remain incurable [3]. In addition, almost all patients receiving androgen ablation therapy as the primary treatment for metastatic PCa end with developing castration-resistant prostate cancer (CRPC) which fails most of the standard treatment [4, 5]. Therefore, there is a great need for verifying novel specific targets and identifying better therapeutic strategies against CRPC.

The ubiquitin-proteasome system (UPS) and the autophagy-lysosome pathway are two major protein degradation systems in all eukaryotic cells. The UPS, a highly specific and selective route for cellular protein degradation, controls the fate of most proteins by striking a dynamic balance between ubiquitination and deubiquitination of protein substrates [6, 7]. Deubiquitinating enzymes (DUBs) regulate multiple cellular processes, including cell cycle control, DNA damage response and repair, apoptosis, chromatin modification, and various signal transduction pathways [6, 8, 17]. Thus, DUBs have recently been identified as novel targets of anti-cancer therapy. The current count of DUBs encoded by the human genome approximately amounts to 98, classified into six families according to their catalytic and structural features. In mammalian cells, three different DUBs are associated with the 19S regulatory particle of the proteasome: USP14, UCHL5/UCH37 and Rpn11. Both USP14 and UCH37/UCHL5 are cysteine isopeptidases that cleave distal polyubiquitin chains and are suggested to hinder substrate degradation. Additionally, RPN11/POH is a Zn^2+^ -dependent protease of the JAMM family resided within the lid of the 19S regulatory particle [9–12]. Notably, these DUBs are often overexpressed in several types of carcinoma cells, implicating their potential to become therapeutic targets for cancer treatment [13–15].

A recent study described b-AP15 as an inhibitor of the USP14 and UCHL5 DUBs of the 19S regulatory particle. In contrast to inhibitors of the 20S proteasome, b-AP15 blocks the deubiquitylating activity of USP14 and UCHL5, which are associated with the 19S regulatory particle, without affecting proteolytic activities of the 20S proteasome [8, 10, 11]. Although b-AP15 is able to induce apoptosis in several kinds of carcinoma [8, 9, 15, 16], its effect on prostate cancer remains unknown. Here, we provide evidence that treatment with b-AP15 inhibits the growth of PCa cells and enhances apoptosis of both androgen receptor-dependent and -independent prostate cancer cells, both *in vitro* and *in vivo*, associated with induction of caspase activation, ER stress and generation of reactive oxygen species (ROS).

## RESULTS

### b-AP15 treatment suppressed cell proliferation and colony formation in prostate cancer cells

**Figure 1:**
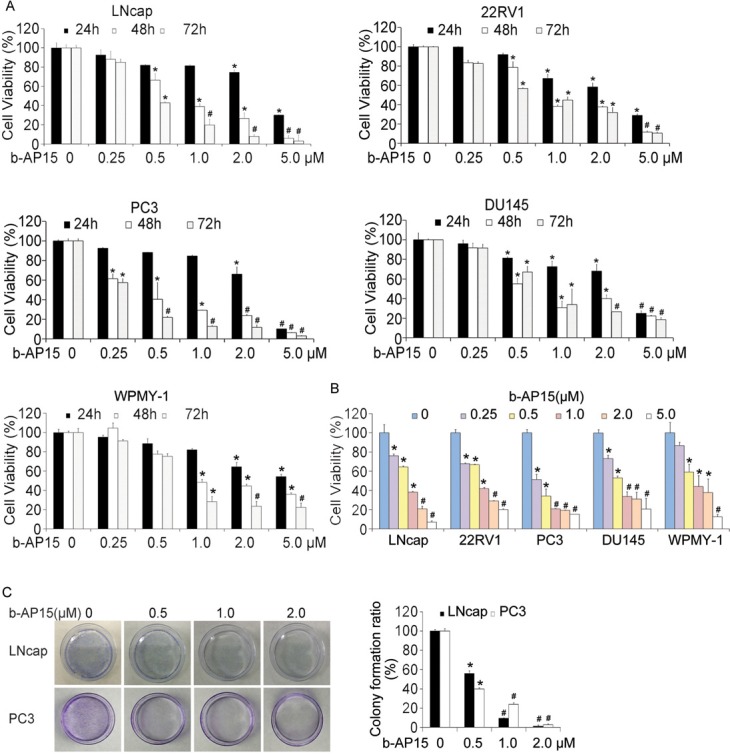
b-AP15 treatment dose-dependently reduced cell viability of PCa *in vitro* **(A)** and **(B)** LNCaP, 22Rv1, PC-3, DU145 and WPMY-1 were treated with increasing concentrations of b-AP15 (0 to 2μM) for 24, 48 and 72 h, and cell viability was detected by MTS assay and CCK8 assay. Mean ± SD (n = 3). *P < 0.05, ^#^*P* < 0.01 *versus* each vehicle control. **(C)** LNCaP and PC-3 cells exposed to b-AP15 (0 to 2μM) for 48 h were suspended in 30% agarose for 7 days, representative images and quantification of colony formation are shown.

Previous studies have shown that treatment with b-AP15 induces tumor cell apoptosis in a series of cancer cell lines [16]. Here, we further confirmed this effect in several prostate cancer cells and a human prostate stromal cell by the MTS assay evaluating cell viability. Increasing concentrations of b-AP15 (0–5μM) treatment for 24 h, 48 h and 72 h inhibited the proliferation of LNCaP, 22Rv1, PC-3, DU145 and WPMY-1 cells indicating no obvious selectivity between cancer cells and normal cells (Figure 1A). We further analyzed these cells under the 48h-exposue to b-AP15 and obtained the IC50 values of 0.762, 0.858, 0.378, 0.748 and 0.958μM, in LNCaP, 22Rv1, PC-3, DU145 and WPMY-1 cells respectively. Consistent with MTS assay, another cell viability assay, the CCK-8 assay, also revealed that b-AP15 significantly suppressed cell proliferation (Figure 1B). Additionally, the dose-dependent suppression of long-term colony formation in LNCaP and PC-3 cells exposed to b-AP15 for 7 days was observed (Figure 1C). These data demonstrated the anti-cancer effect of b-AP15 against PCa cells.

### b-AP15 induces cell cycle arrest *in vitro*

**Figure 2:**
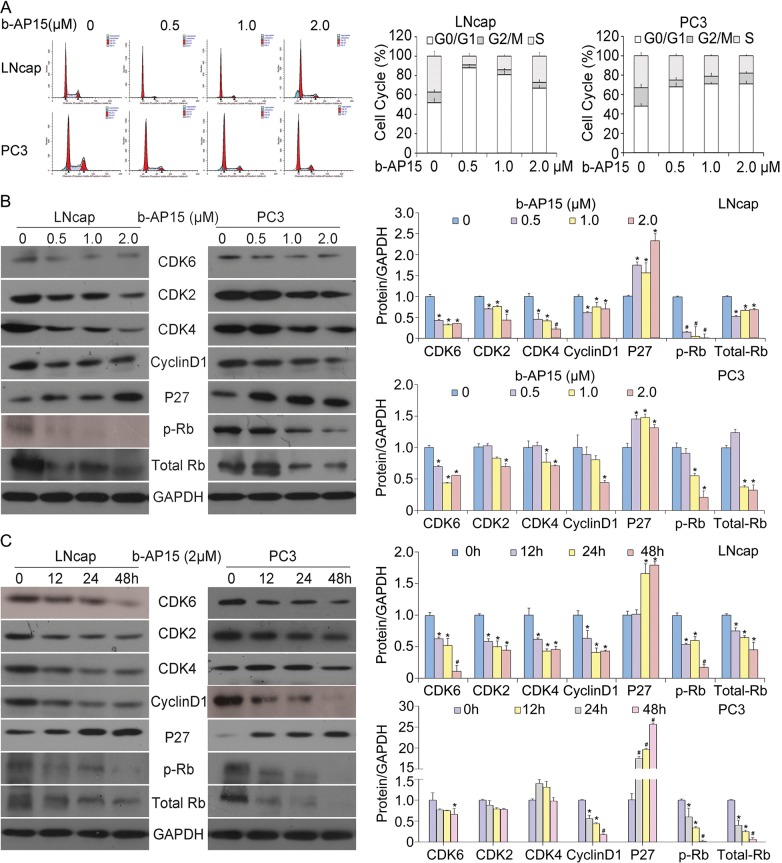
b-AP15 treatment induced G0/G1 cell cycle arrest in LNCaP and PC-3 cells **(A)** LNCaP and PC-3 cells were treated with the indicated concentrations of b-AP15 (0 to 2μM) for 24 h, harvested and fixed in 70% ethanol. After washing with phosphate-buffered saline, cells were stained with PI, and DNA content of cells was analyzed with flow cytometry. **(B)** LNCaP and PC-3 cells were exposed to the indicated concentrations of b-AP15. Protein lysates were subject to western blot analysis for CDK6, CDK4, CDK2, cyclinD1, p27, phosphor-Rb and Rb. GAPDH was used as a loading control. And quantifications of band density are shown. *P<0.05 or ^#^*P*<0.01 *versus* control group. **(C)** LNCaP and PC-3 cells were exposed to b-AP15 2μM for different durations. Protein lysates were subject to western blot analysis using antibodies as mentioned above. GAPDH was used as a loading control. And quantifications of band density are shown. *P<0.05 or ^#^P<0.01 *versus* control group.

The antitumor effect depicted above led us to investigate the mechanism of b-AP15 inhibiting PCa cell growth. Given that DUBs play a pivotal role in determining the fate of proteins regulating the cell cycle [18], we sought to study the effect of b-AP15 on cell cycle regulation in androgen receptor-dependent and LNCaP androgen receptor–independent PC-3 cell lines. Cells were exposed to various concentrations of b-AP15 (0, 0.5, 1, 2μM) and subsequently subject to cell cycle analysis; and we found that increasing dosage of b-AP15 dramatically induced G0/G1 cell cycle arrest in both cell lines at 24 h (Figure 2A). To further determine changes in the levels of proteins that regulate cell cycle, we performed western blot analyses for several proteins that are related to G1-S phase transition. Consistent with the cell cycle arrest, We found that b-AP15 significantly decreased the expression of cyclin D1, CDK6, CDK4, CDK2 and phosphorylation/inactivation of Rb-marker proteins from G1 to S phase, and up-regulated the expression of p27, which is a known tumour suppressor *via* inhibiting the cyclin-CDK function (Figure 2B and 2C).

### Induction of caspase-dependent apoptosis by b-AP15

**Figure 3:**
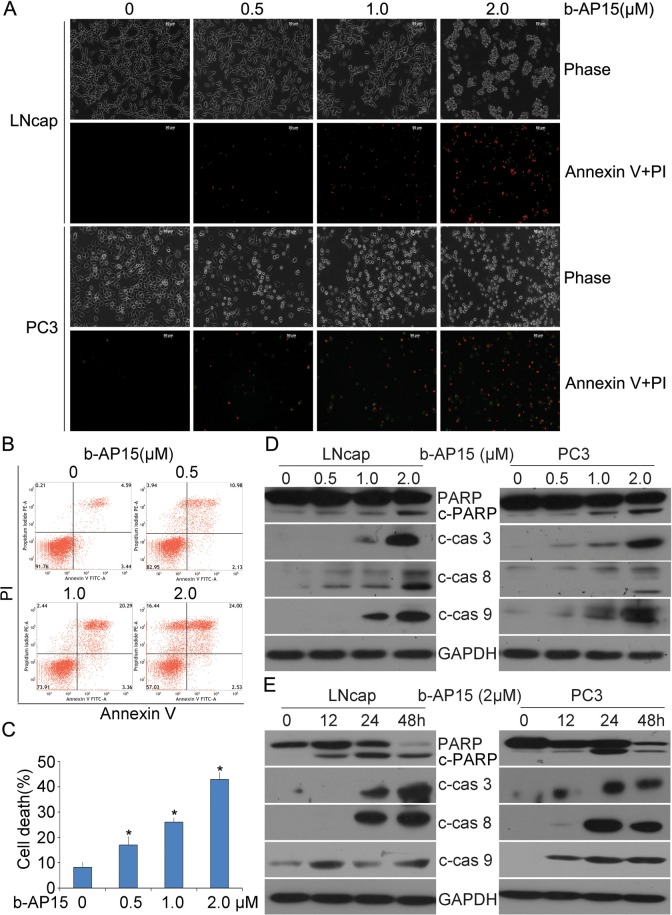
b-AP15 treatment induced cancer cell death **(A)** LNCaP and PC-3 were treated with different doses of b-AP15 for 48 h, then apoptotic cells were detected by Annexin V-FITC / Propidiumiodide (PI) double staining, and the stained cells were recorded using an inverted fluorescence microscope (AxioObsever Z1, Zeiss, Germany). The representative emerged images are shown. **(B, C)** PC-3 were seeded in 6-well plates and exposed to b-AP15 for 48 h. The cultured cells were collected and stained with Annexin V FITC/propidium iodide (PI), followed by flow cytometry analysis. The representative images **(B)** and summary of cell death **(C)** are shown. Mean ± SD (n = 3). *P < 0.05 versus vehicle control. **(D, E)** LNCaP and PC-3 were treated with b-AP15at the indicated doses (0 to 2μM) for the indicated durations (0-48 h). Total proteins were extracted from the cultured cells and subject to western blot analysis for cleaved caspase-3, -8 and -9, and PARP. GAPDH was used as a loading control.

In attempt to evaluate whether the exposure of b-AP15 (0–2 μM) to human prostate cancer cells induces cell death, Annexin-V FITC and propidium iodide (PI) double staining were applied on LNCaP and PC3 living cells and detected with fluorescence microscopy (Figure 3A) or quantified by flow cytometry (PC-3 cells; Figure 3B and 3C). It was found that the Annexin-V/PI-positive population significantly increased and the morphological changes of apoptosis appeared in b-AP15 treated cells. Further, caspase activation is a major step of the apoptosis pathways in multiple cancer cells [19], and our data show that b-AP15 elicited significant activation of caspase-3, -8, -9 in LNCaP and PC-3 cells, together with the cleavage of PARP, a hallmark of apoptosis (Figure 3D and 3E). These results indicate that b-AP15 activates multiple caspase proteins and induces apoptosis in PCa cells.

### Induction of apoptosis by b-AP15 is associated with mitochondrial dysfunction

**Figure 4:**
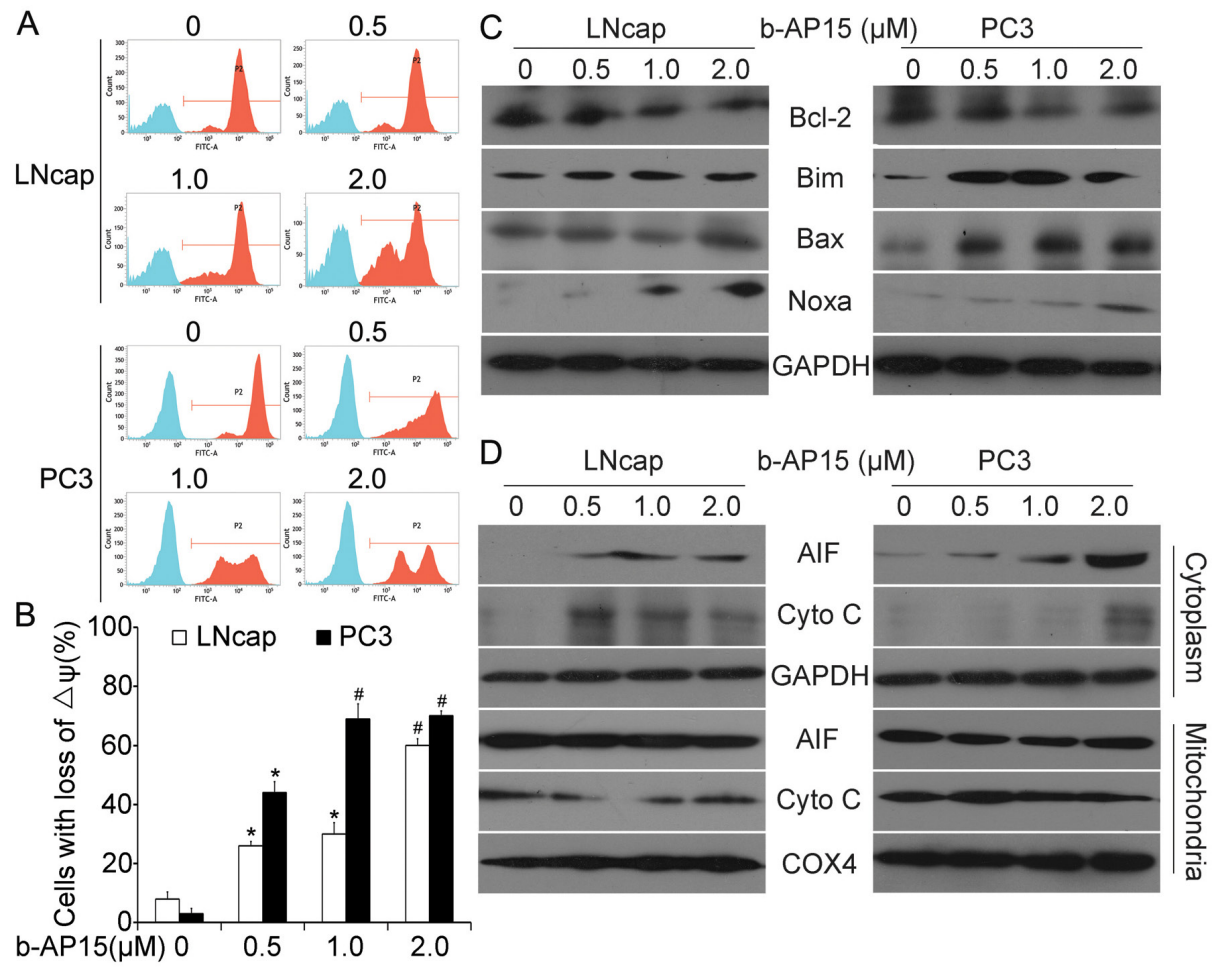
b-AP15 treatment altered the expression of mitochondria-related proteins **(A and B)** LNCaP and PC-3 were exposed to increasing concentration of b-AP15 (0 to 2μM) for 48 h. Mitochondrial membrane potential was detected by rhodamine-123 staining and flow cytometry analysis. The proportion of cells with loss of ΔΨ was shown. Graphs represent data from three independent experiments. Mean ± SD (n = 3). *P < 0.05, ^#^*P* < 0.01 compared with vehicle control. **(C and D)** Cancer cells were treated as in (A), total Bcl-2, Bim, Bax and Noxa were detected by western blot analysis **(C)**. AIF and cytochrome C in the cytoplasm and mitochondria were analyzed with western blot **(D)**.

Mitochondria exert central and multifunctional roles in cancer metabolism and modulation of apoptotic pathways [20, 21]. In addition to triggering apoptotic signals, we also observed that b-AP15 treatment led to the loss of mitochondrial membrane potential, as detected by rhodamine-123 staining and flow cytometry (Figure 4A and 4B). BCL-2 family proteins, the major regulators of cell survival and death, play pivotal roles in mitochondrial metabolism [22–24]. To further investigate the mechanism by which b-AP15 causes apoptotic cell death, the expression of several cardinal apoptosis-related proteins of BCL-2 family members was measured. As shown in Figure 4C, b-AP15 triggered a remarkable decline in the expression of anti-apoptotic protein (Bcl-2) in LNCaP and PC-3 cells. Meanwhile, a significant increase of the pro-apoptotic proteins (Bim, Bax, Noxa) were also observed.

Moreover, when looking at the cytosolic and mitochondrial fractions of LNCaP and PC-3 cells, we found that 48 h exposure to escalating doses of b-AP15 increased the level of pro-apoptotic factors (cytochrome C) and apoptosis-inducing factor (AIF). Taken together, these findings support the argument that b-AP15 induces apoptosis through the mitochondrial pathway by down-regulating the integrity of mitochondrial membranes, release of cytochrome C and AIF (Figure 4D).

### N-acetyl-cysteine(NAC) reversed b-AP15-induced ROS generation and apoptosis

**Figure 5:**
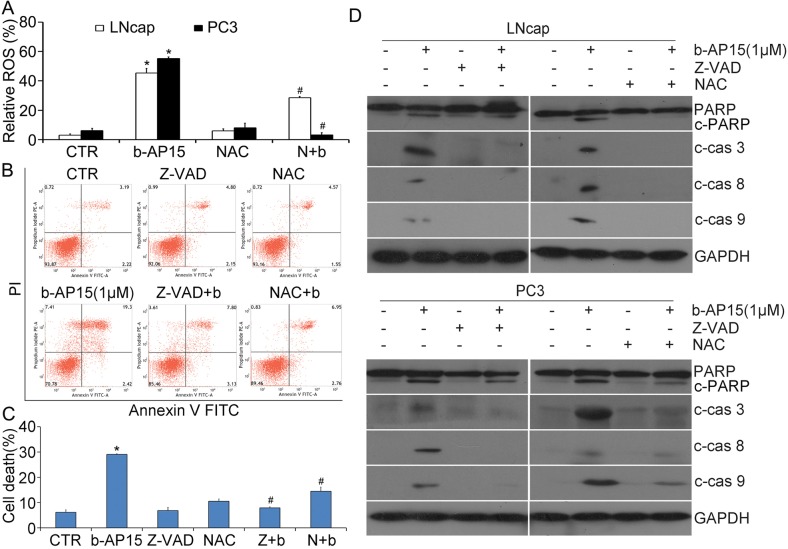
ROS generation was increased by b-AP15 and induction of apoptosis by b-AP15 was inhibited by z-VAD-FMK and NAC **(A)** LNCaP and PC-3 cells were treated with b-AP15 (1μM) in the absence or presence of 5 mM NAC for 12 h. ROS generation was detected by flow cytometry after the cells being stained with DCFH-DA. Relative ROS was shown. Mean ± SD (n = 3). *P < 0.05 *vs* vehicle treatments. ^#^*P* < 0.05 *vs* b-AP15 treatments. **(B)** and **(C)** PC-3 cancer cells were treated with b-AP15 in the absence or presence of z-VAD-FMK or NAC for 12 h. The treated cells were collected to stain with Annexin V FITC/PI, followed by flow cytometry analysis. Data of three independent experiments are summarized and shown. Mean ± SD (n = 3). *P < 0.05 versus vehicle control; ^#^*P* < 0.05 compared with b-AP15 treatments. **(D)** LNCaP and PC-3 were exposed to b-AP15 in the absence or presence of z-VAD-FMK (50 μM) or NAC (5 mM) for 12 h. PARP cleavage, cleaved caspase-3, -8 and -9 were detected by western blot analyses. Representative images of independent experiments are shown. GAPDH was used as a loading control.

Compared with the normal cells, cancer cells become more sensitive to ROS induction of cell death [25–28]. Not surprisingly, experiments using the fluorescent ROS probe 2′,7′-dichlorofluorescin diacetate (DCFH-DA) in LNCaP and PC-3 cells unveiled that b-AP15 triggers higher levels of ROS, which was reversed by pretreatment of antioxidant (NAC)(Figure 5A). The Annexin-V FITC and propidium iodide (PI) double staining revealed that both Z-VAD-FMK and NAC were able to prevent b-AP15 treatment from inducing cell death (Figure 5B and 5C).

To further demonstrate that the pro-apoptotic effects of b-AP15 is owing to the activation of caspase in LNCaP and PC-3 cells, caspase expression and activation was detected by western blot analysis after pre-treatment of the pan-caspase inhibitor Z-VAD-FMK and antioxidant (NAC). In the experiments shown in Figure 5D, the activation of caspase-3, -8 and -9 and the cleavage of PARP by b-AP15 were significantly attenuated by NAC or Z-VAD-FMK. Taken together, these data suggest that the inhibtion of growth and induction of cell death by b-AP15 are mediated by ROS generation and the activation of pro-apoptotic caspases.

### b-AP15 triggers accumulation of ubiquitinated proteins (Ub-prs), ER stress and suppression of androgen receptor (AR)

**Figure 6:**
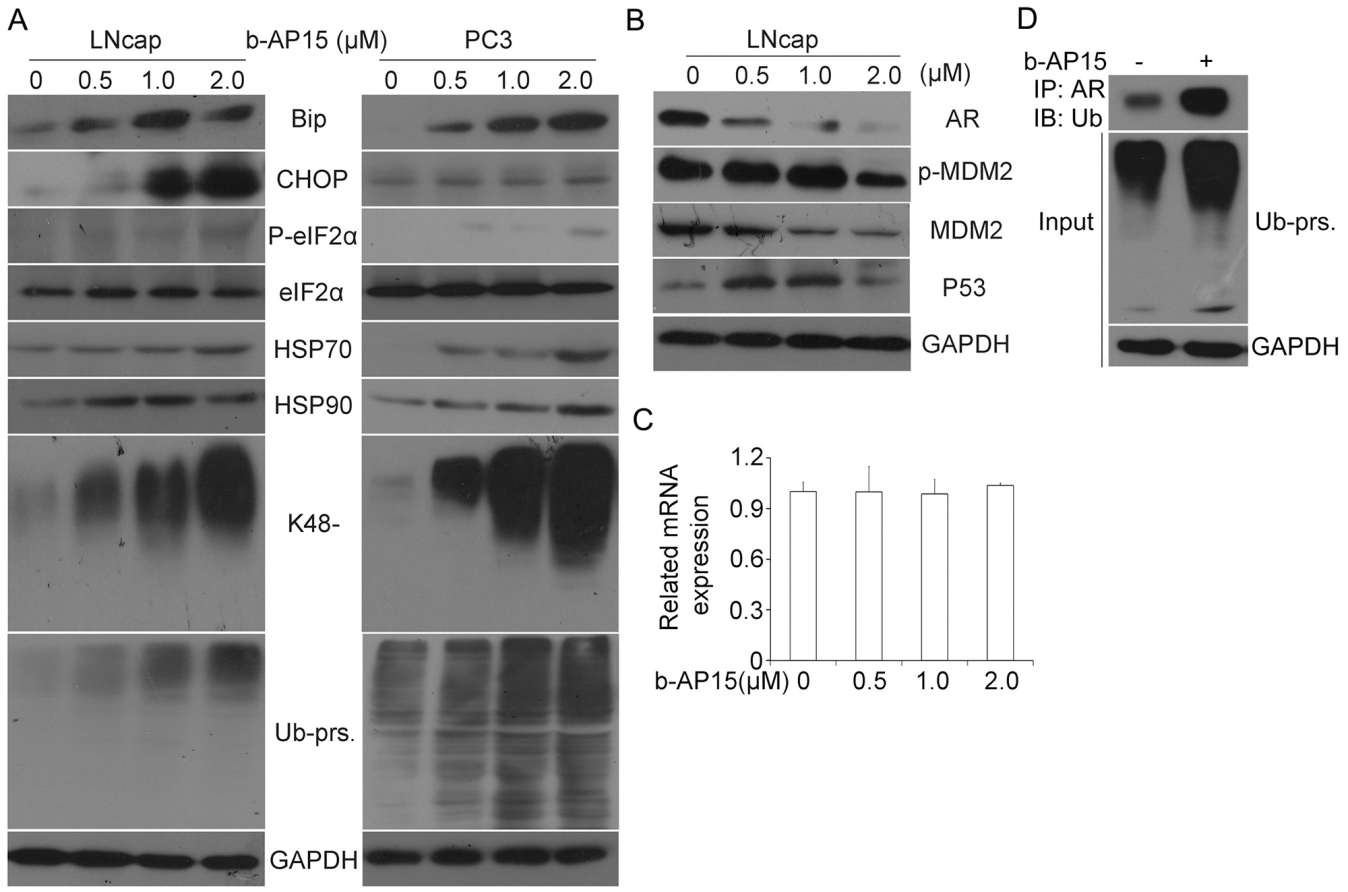
b-AP15 treatment induced accumulation of ubiquitinated proteins, ER stress and AR inhibition **(A)** LNCaP and PC-3 cells were treated with 0 to 2μM b-AP15 for 24 h. Proteins extracts from the treated cells were subject to western blot analysis for BiP, CHOP, P-eIF2α, eIF2α, HSP70, HSP90, K48-linked ubiquitin,and total ubiquitinated proteins (Ub-prs). GAPDH blot was used as a loading control. **(B)** LNCaP cells were exposed to the indicated concentrations of b-AP15. Total proteins were extracted and subject to western blot analysis for AR, MDM2 and ser166-phosphorylated MDM2 (phospho-MDM2), and p53. GAPDH was used as a loading control. **(C)** LNCaP cells were exposed to b-AP15 1μM for 24 h. Total RNA were extracted and subject to RT-qPCR analysis. GAPDH was used as an internal control. Three independent experiments were performed. Mean ± SD (n = 3). **(D)** LNcap cells were exposed to b-AP15 1μM for 24 h, immunoprecipitated with AR beads and immunoblotted with antibodies to Ub-prs. Cells were treated with MG132 (10 μM) for 6 h before being harvested.

The effect of b-AP15 on ubiquitin-dependent protein degradation was evaluated in the prostate cancer cell lines LNCaP and PC-3. In our experiments, incremental concentrations of b-AP15 (0–2μM) were applied to the prostate cancer cells for 24 h; the effect on cellular accumulation of Ub-prs was measured with western blot analysis. As shown in Figure 6A, b-AP15 treatment induced accumulation of both total and K48-linked (K48-) ubiquitin proteins in LNCaP and PC-3 cell lines. As reported, b-AP15 is able to induce the ER stress response [8, 9, 15], Consistently, here we found that b-AP15 treatment markedly increased the protein expression of heat shock protein 70 (HSP70), HSP90 as well as other ER stress related proteins, including Bip, CHOP, and phosphorylated eIF2α (p-eIF2α).

We have previously reported that USP14 regulates prostate cancer proliferation by deubiquitinating and stabilizing androgen receptor [29]. Consistent with b-AP15 being an inhibitor of USP14 and UCHL5, the expression of total androgen receptor and MDM2 were significantly suppressed in LNCaP cells, while phosphorylation of MDM2 and tumor suppressor protein p53 increased, after b-AP15 treatment (Figure 6B). To test whether suppression of AR result from protein modification, we utilized RT-qPCR to measure the mRNA expression of AR and found that the mRNA level was not changed by b-AP15 treatment (Figure 6C). In contrast, when we immunoprecipitated AR and immunoblotted with antibodies against ubiquitin at the presence of MG132, we found a greater accumulation of ubiquitinated-AR in LNCaP cells when exposed to b-AP15 (Figure 6D). These results suggest that b-AP15 destabilizes AR proteins *via* suppressing the deubiquitination of AR.

### Anti-cancer activity of b-AP15 *in vivo*

**Figure 7:**
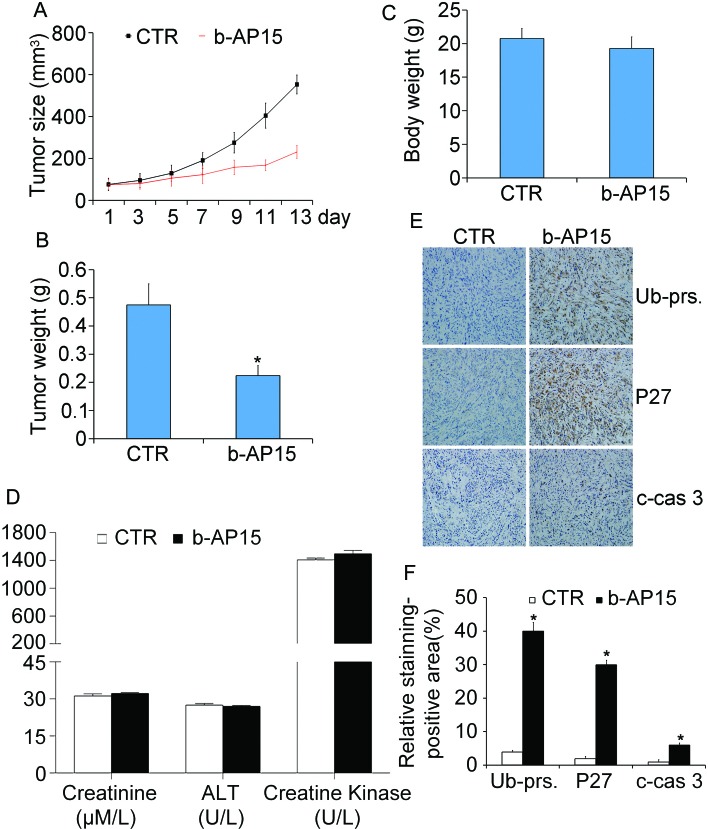
b-AP15 treatment inhibited tumor growth *in vivo* BALB/c nude mice bearing PC-3 xenografts were treated with b-AP15 (5mg/kg/2d,i.p.) for 14 days. Tumor size **(A)**, tumor weight **(B)**, and body weight **(C)** are shown. Mean ± SD. *P < 0.05 *versus* vehicle treatment. **(D)** CK, ALT and creatinine from mouse serum were measured at the end of treatment. Data shown are mean±SD. **(E)** Representative micrographs of immunohistochemical staining for total ubiquitinated proteins (Ub-prs), proteasome substrate protein (p27), and cleaved caspase-3 in nude tumor tissues from mice bearing PC-3 tumors treated with either vehicle or b-AP15 (200 ×) and the expression was quantified **(F)**. *P < 0.05 *versus* vehicle treatment.

To further evaluate the antitumor effect of b-AP15 *in vivo*, nude mouse xenograft models of human prostate cancer cell line PC-3 were established and treated with b-AP15. Compared with the control group, we found that the tumor size and tumor weight of the group treated withb-AP15 were strikingly reduced, as shown in Figure 7A and 7B. Importantly, there was no significant difference in body weight of the nude mice between the two groups (Figure 7C). Additionally, we used the immunohistochemistry staining to analyze the effect of b-AP15 on proteasome function *in vivo*. We found that the levels of proteasome substrates, including total ubiquitin proteins and p27, were markedly increased in the b-AP15-treated tumor tissues, suggesting an inhibition of deubiquitination. Moreover, the immunohistochemistry also revealed that cleaved caspase-3 was also increased in b-AP15 treated tumor cells (Figure 7E and 7F). Shown in Figure 7D, there were no significant dysfunctional difference of heart, liver and kidney compared with the control group, meaning that the toxic impact by b-AP15 can be effectively controlled. In summary, these data indicate that b-AP15 induces proteasome inhibition, caspase activation and suppression of tumor growth *in vivo*, consistent to the results *in vitro*.

## DISCUSSION

Recent evidence suggests that deubiquitinases (DUBs) play critical roles in proteasomal protein degradation which has been well-established to be involved in cancer progression and, as such, becomes a novel target for anticancer strategy [10]. Although the USP14/UCHL5 DUB inhibitor b-AP15 induced anti-tumor response in several types of malignant tumor including colorectal cancer, myeloma and breast cancer [8, 9, 15], its effect on human prostate cancer cells, especially the castration-resistant prostate cancers (CRPC), is undefined yet. Despite a few therapeutic options approved for clinical use, CRPC are virtually an incurable disease which turns to be a great challenge for the current development of clinical therapy [3]. Hence, developing a novel therapeutic approach for the advanced disease of prostate cancer is a crucial urgency.

Our observations suggest that b-AP15 treatment leads to an inhitbion of cell proliferation in a dose- and time-dependent manners in both AR-responsive and -irresponsive PCa cells. Moreover, we found that the expression of cyclin D1, CDK4, CDK6, CDK2, and phospho-Rb were significantly down-regulated whereas the level of p27 increased after treatment, together with a cell cycle arrest. There are two protein families involved in cell cycle regulation, the cyclin-dependent protein kinases (CDKs) and the cyclins [30]. During early–mid G1 phase of the cell cycle, cyclin D associates with CDK4 and CDK6, driving orderly cell cycle progression although additionally, CDK2 regulates G1 to S phase transition. Besides, Rb protein acting as a potent tumor suppressor gene plays a basic role throughout the cell cycle progression and exerts function of restricting proliferation when it was hypo-phosphorylated (active) in G1 phase. Another protein like p27, serving as a CDK inhibitor (CKI), potently blocks the G1 to S phase transition *via* binding with Cyclin/Cdk [30–33].

In this study, b-AP15 treatment contributes to the apoptosis in LNCaP and PC-3 cells *via* both extrinsic and intrinsic pathways as shown by the activation of caspase-8, caspase-9 and caspase-3, as well as PARP cleavage which can be blocked by pan-caspase inhibitor z-VAD-FMK. The caspases, a family of cysteine proteases, have been unequivocally linked to the regulation of apoptosis which can be stimulated by extrinsic and intrinsic (mitochondrial) pathways. It can be divided roughly into two major groups, including the initiators such as caspase-2, -8, -9, and -10; and the effectors such as caspase-3, -6, -7, and -14 [34]. Since caspase-8 was activated in the extrinsic pathway and caspase-9 in the intrinsic pathway which is regulated by mitochondria, executioner caspase-3 is subsequently activated and cleaves a variety of target substrates, such as poly (ADP-ribose) polymerase (PARP) [35–38]. Additionally, mitochondria play essential roles in tumor growth and progression *via* regulating cell death by apoptosis and necrosis [20]. Alteration of mitochondrial membrane potential and release of pro-apoptotic factors result from the apoptotic signals, processes that are more germane to Bcl-2 family proteins [24]. We found that b-Ap15 triggered down-regulation of anti-apoptotic proteins (e.g., Bcl-2) and the increase of pro-apoptotic proteins such as Bim, Noxa and Bax, together with the loss of mitochondrial membrane potential (ΔΨ) and the release of cytochrome C (Cyto C) and apoptosis-inducing factor (AIF) from the mitochondria to cytoplasm [39, 40], indicating that the activation of apoptosis *via* the intrinsic pathway is a critical responder of b-AP15 treatment.

Oxidative stress is often highly generated by antitumor therapy in several cancer cells [41]. Although ROS may be involved in multiple steps of tumorigenesis, malignant cells seem to be more vulnerable to oxidative stress-induced cell death compared to the benign ones [26]. b-AP15 was previously reported to induce ROS accumulation in human colorectal cancer HCT116 cells [9]; however, whether this occurs in the prostate cancer remains unknown. Here, we found that exposure of b-AP15 induces higher ROS in both LNCaP and PC-3 cells. Importantly, NAC, a classical ROS scavenger, diminished the ability of b-AP15 to induce apoptosis, as evidenced by remarkable attenuation of caspases-3 -8 -9 and PARP cleavage. These data confirm that oxidative stress plays a pivotal role in b-AP15-induced apoptotic cell death in prostate cancer.

Intriguingly, we not only found that b-AP15 induced accumulation of both total and K48 linked Ub-prs in PCa cells, but further characterized the involvement of ER stress in b-AP15-induced cell death. As shown, the expression of HSP70 and HSP90 and a stack of ER stress related proteins such as Bip, CHOP and p-eIF2α were elevated. HSP70 and HSP 90 are two primary proteins among the heat shock proteins (HSPs) which play significant roles in cell homeostasis and cytoprotection under various stress situations [42]. When cells are undergoing stress a heat shock response (HSR) is aroused. The induction of several heat shock proteins including HSP70 and HSP27 shared the phenomenon, and is mediated at the transcriptional level by heat shock factor 1 (HSF-1), which serves as the “conductor” of the HSR. Similar effect was observed in PCs cells after treating with b-AP15. In normal situations, HSF-1 is bound to HSP90 to form inactive monomers. When it comes to stress situations, this complex dissembles and HSF-1 is released, hyperphosphorylated and attaches to DNA in the form of an active trimer, contributing to the transcription of HSP genes [43]. Generally, the Unfolded Protein Response (UPR) pathways would be further activated, coordinating adaptive and apoptotic responses to the stress in the ER [44]. BiP/GRP78 is an ER chaperone which belongs to the heat-shock protein family. Under abnormal conditions, BiP/GRP78 release subsequently invokes the induction of protein kinase RNA-like endoplasmic reticulum kinase (PERK). As a result, the level of phosphorylated eIF2α is significantly increased, followed by the translation of ATF4 which then promotes expression of pro-death transcription factor C/EBP homologous protein (CHOP) [45, 46]. These results indicate that the induction of ER stress at least partially contributes to b-AP15-induced cell apoptotic death. Furthermore, we found that b-AP15 triggered down-regulation of AR and MDM2 as well as an increase of phosphorylated MDM2 and p53 in LNCaP cells. MDM2 is a E3 ligase of AR and p53 [29, 47, 48], so the increase in p53 protein levels but not the decrease of AR in the b-AP15 treated cells is consistent with the decreased MDM2. The total AR expression was decreased by b-AP15, which may result from the major impact of USP14 suppression [29], suggesting that USP14 is critical for the stabilization of AR. Nevertheless, there is great possibility that the downregulation of AR by b-AP15 is mediated by enhanced proteasome-mediated degradation because (1) when proteasome was inhibited, b-AP15 treatment failed to down-regulate AR but was capable to increase ubiquitinated AR (Figure 6D) and (2) b-AP15 at a dosage sufficient to downregulate AR proteins did not alter the levels of steady state AR mRNA (Figure 6C). Notably, AR is critical for PCa progression and considered as a key factor for delaying the progression from androgen-dependent prostate cancer to castrate-resistant prostate cancer [49]. Therefore, exploring the effect of b-AP15 on AR stabilization will help identify novel agents to block AR signaling or down-regulate AR expression, delaying the PCa progression through targeting AR.

The effect of b-AP15 treatment on prostate cancer xenografts highly resembles our *in vitro* findings. We have found that b-AP15 treatment effectively suppressed the growth of prostate cancer xenografts in nude mice, which is associated with accumulation of proteasome substrates (e.g., total ubiquitinated proteins and tumor suppressor p27) as well as elevated levels of cleaved caspase 3. Notably, together with the antitumor and pro-apoptotic activity of the treatment, no significant reduction of body weight was discernible, implicating that the treatment is well tolerated. Intriguingly, b-AP15 was able to eliminate prostate cancer cells and normal cells *in vitro*, but does no harm to the function of heart, liver and kidney on tumor-bearing mice. Although further toxicity evaluation is required, our study indicates that b-AP15 represents a promising new drug candidate for further pre-clinical and clinical therapeutic development in battling castrate-resistant prostate cancer (CRPC).

## MATERIALS AND METHODS

### Materials

b-AP15 was obtained from Selleck Chemicals (Houston, TX, USA) and dissolved in DMSO at a stock concentration of 10 mM, aliquoted and stored at −80°C. Other agents are N-acetyl-L-cysteine (NAC, Sigma-AldrichInc., St. Louis, MO); pan caspase Inhibitor Z-VAD-FMK (EnzoLife Sciences International, Inc, Plymouth Meeting, PA). Antibodies (Abs) used in this study were purchased from following sources: anti-CDK2, CDK4, CDK6, anti-cyclinD1, anti-p27, anti-PARP, anti-Bcl-2 (50E3), anti-Bim (Y36), anti-Noxa (D8L7U), anti-BIP (C50B12), anti-CHOP (L63F7), eIF2α, phospho-eIF2α (Ser51), anti-HSP70, anti-HSP90, anti-AIF, cytochrome C and anti-Phospho-MDM2 (Cell Signaling Technology, Beverly, MA, USA); anti-Rb, phospho-Rb (S780), anti-cleaved caspase-8 (Cleaved Asp384) (Assay biotechnology Company, Inc); anti-cleaved caspase-9 p35 (D315), anti-cleaved caspase-3 (p17), anti-AR, anti-MDM2 and anti-GAPDH (Bioworld Technology, Inc). anti-p53(Abcam), anti-ubiquitin (P4D1), anti-K48-linked tetra-ubiquitin, Bax (B-9) (Santa Cruz Biotechnology, Santa Cruz, CA); MTS assay (CellTiter 96 Aqueous One Solution reagent) was purchased from Promega Corporation (Madison, WI, USA). CCK-8 assay kit, PI and Annexin V-FITC apoptosis Detection Kit, DCFH-DA and cell apoptosis Rhodamine 123 Detection Kit were purchased from Keygen Company (Nanjing, China). Dynabeads antibody coupling kit was from Life technologies.

### Cell lines and cell culture

Human prostate cancer cell lines LNCaP, 22Rv1, PC-3, DU145 and human prostate stromal cell line WPMY-1 were purchased from American Type Culture Collection (Manassas, VA, USA). LNCaP and 22Rv1 cells were grown in RPMI 1640, PC3, DU145 were in Hyclone DMEM/F-12 and WPMY-1 were in DMEM supplemented with 10% FBS. Cultured cells were maintained at 37°C and 5% CO2 [29].

### Cell viability assay

MTS assay (CellTiter 96 Aqueous One Solution reagent) was used to test cell viability as we previously reported [50]. Briefly, LNCaP, 22Rv1, PC-3, DU145 and WPMY-1 cells were seeded at 2500 cells per well in a 96-well plate. After 24 h, cells were treated with b-AP15, followed by continuous incubation for 24, 48, or 72 h. 20 μl MTS was directly added to each well and the incubation was continued for an additional 3 h. The absorbance density was read on a 96-well plate reader at wavelength 490 nm. IC_50_ values were calculated. The CCK8 assay was used to determine cell viability as well. The absorbance at 450 nm was measured with a Quant Universal Microplate Spectrophotometer (BioTek, Winooski, VT, USA). Three independent experiments were performed and the data were presented as the mean ± SD.

### Cell death assay

Apoptosis was determined by flow cytometry using Annexin V-fluoroisothio-cyanate (FITC)/PI double staining according to previous description [51]. Cells were seeded into 6-well plates and treated with b-AP15. Cells were harvested by trypsinization and washed with 4°C PBS twice then resuspended with the binding buffer, followed by Annexin V-FITC incubation for 15 min and PI staining for another 15 min in dark. The double-stained cells were also imaged with an inverted fluorescence microscope equipped with a digital camera (AxioObsever Z1, Zeiss, Germany).

### Colony formation assay

This assay was performed as we previously described [52]. LNCaP and PC-3 cells exposed to b-AP15 for 48 h were suspended in 30% agarose supplemented with 20% FCS and 50% RPMI-1640 medium (for LNCaP cells) or (for PC-3) in 60mm dishes and cultured in an atmosphere of 5% CO 2 for 7 to 10 days, then stained with 0.3% crystal violet solution. The colonies> 60 μm were counted under a light microscope. The experiments were done in triplicate.

### Western blot and co-IP analysis

For IP and western blot, dynabeads m-270 Epoxy (Invitrogen) coupled with antibodies were prepared and then cell lysates were added, and the antibodies-lysate mixtures were rotated at4°C for 1 h. Immunocomplexes separated from dynabeads were washed with lysis buffer and then suspended with SDS blue loading buffer. To detect ubiquitinated proteins, lysis was performed under 80°C for 10 min [29]. Western blot was performed as we described previously [53]. In brief, an equal amount of total protein extracted from cultured cells were fractionated by 12% SDS-PAGE and transferred to polyvinylidenedifluoride (PVDF) membranes. The blots were blocked with 5% milk for 1 h. Primary antibodies and horseradish peroxidase conjugated appropriate secondary antibodies were used to detect the designated proteins. Blots were reacted to the ECL detection reagents and exposed to X-ray films (Kodak, Japan).

### Mitochondrial membrane integrity measurement

The mitochondrial membrane potential of b-AP15 and untreated cells was assayed by using Rhodamine-123 staining as we previously reported. Cells were treated with various concentrations of b-AP15 for 48 h and stained with 1μM of rhodamine-123 for30 minat 37°C. Following the staining, the cells were washed with 4°C PBS and then harvested for flow cytometry analysis. Mean values and standard deviations were calculated from triplicates [54].

### Measurement of ROS generation

ROS production was detected as previously reported [55]. Cancer cells were seeded in a 6-well plate, treated with b-AP15 for 12 h after adhesion, and then stained using 10μM of DCFH-DA for 20 min at 37°C. Stained cells were washed with 4°C PBS twice and subsequently analyzed through flow cytometer. The fold changes of mean fluorescence intensities were shown in the diagram. Mean values and standard deviations were calculated from triplicates.

### Quantitative real-time polymerase chain reaction (qRT-PCR)

Total RNA was isolated using RNAisoPlus (TaKaRa) according to the manufacturer's instructions. After quantification by spectrophotometry, the first-strand cDNA was synthesized from 500ng of total RNA using the PrimeScript II 1st Strand cDNA Synthesis Kit (TaKaRa, Dalian, China) and random primers. Then 50 ng of total cDNA was use for real-time PCR with the SYBR Premix Ex Taq II Kit (TaKaRa) under the following conditions: 95°C for 30 s, followed by 40 cycles of 95°C for 5 s, 60°C for 30 s. The reaction used the StepOnePlus real-time PCR system (Applied Biosystems Inc., USA). PCR primers are as following. AR: F: 5′-GGTGAGCAGAGTGCCCTATC-3′; R: 5′-GAAGACCTTGCAGCTTCCAC-3′; GAPDH:F:5′- TCCCATCACCATCTTCCA-3′;R: 5′-CATCACGCCACA GTTTCC-3′. After PCR, a melting curve analysis was performed to demonstrate the PCR product specificity. Every sample was analyzed in triplicate. The relative expression level of a target gene was presented as the sample versus the control [29].

### Nude mouse xenograft model

Male Balb/c nude mice of 5 weeks of age were purchased from Guangdong Animal Center and housed in the animal facility of Guangzhou Medical University approved by the Guangdong Animal Center. The mice were housed in barrier facilities with a 12 h light dark cycle, with food and water available ad libitum. Approximately 1×10^6^ of PC-3 cells were inoculated subcutaneously in the left armpit of each mouse. 72h after cell inoculation, mice were randomly divided into 2 groups and treated with either vehicle (DMSO, cremophor, and 0.85% NaCl at 1:3:6 ratio, v:v:v) and b-AP15 (5mg/kg/2d) for totally 14 days. Tumors were measured every other day with use of calipers. Tumor volumes were calculated as previously reported [13]. Tumor xenografts were removed afterwards, weighed, fixed and stored. All experiments were performed in accordance with relevant guidelines and regulations. All animal studies were conducted with the approval of the Institutional Animal Care and Use Committee of Guangzhou Medical University.

### Immunohistochemical staining

Formalin-fixed xenografts were embedded in paraffin and sectioned according to standard techniques as we previously reported [56]. Tumor xenograft sections (4μm) were immunostained using the MaxVisionkit (MaixinBiol) according to the manufacturer's instructions. The primary antibodies were against ubiquitin, p27, and cleaved caspase 3.50 μl MaxVisionTM reagent was applied to each slide. Color was developed with 0.05% diaminobenzidine and 0.03% H_2_O_2_ in 50 mM Tris-HCl (pH 7.6), and the slides were counterstained with hematoxylin. A negative control for every antibody was also included for each xenograft specimen by substituting the primary antibody with preimmune serum.

### Statistical analysis

All experiments were performed at least thrice. Data are mean ± standard deviation (SD). Unpaired Student's t-test or one way ANOVA is used where appropriate for determining statistic probabilities. GraphPad Prism4.0 software (GraphPad Software) was used for statistical analysis. Significance level was set at *p* value below 0.05.
